# Collision Detection of a HEXA Parallel Robot Based on Dynamic Model and a Multi-Dual Depth Camera System

**DOI:** 10.3390/s22155923

**Published:** 2022-08-08

**Authors:** Xuan-Bach Hoang, Phu-Cuong Pham, Yong-Lin Kuo

**Affiliations:** 1 Graduate Institute of Automation and Control, National Taiwan University of Science and Technology, Taipei 106, Taiwan; 2 Center of Automation and Control, National Taiwan University of Science and Technology, Taipei 106, Taiwan

**Keywords:** collision detection, collision isolation, collision identification, parallel manipulator

## Abstract

This paper introduces a Hexa parallel robot and obstacle collision detection method based on dynamic modeling and a computer vision system. The processes to deal with the collision issues refer to collision detection, collision isolation, and collision identification applied to the Hexa robot, respectively, in this paper. Initially, the configuration, kinematic and dynamic characteristics during movement trajectories of the Hexa parallel robot are analyzed to perform the knowledge extraction for the method. Next, a virtual force sensor is presented to estimate the collision detection signal created as a combination of the solution to the inverse dynamics and a low-pass filter. Then, a vision system consisting of dual-depth cameras is designed for obstacle isolation and determining the contact point location at the end-effector, an arm, and a rod of the Hexa robot. Finally, a recursive Newton-Euler algorithm is applied to compute contact forces caused by collision cases with the real-Hexa robot. Based on the experimental results, the force identification is compared to sensor forces for the performance evaluation of the proposed collision detection method.

## 1. Introduction

A six-degree-of-freedom parallel structure called the Hexa parallel robot was introduced by Pierrot et al. in [1,2]. Despite its complicated kinematics structure and limited workspace, the Hexa robot has remarkable advantages over the serial robot: high accuracy, high stiffness, high speed, and high carrying capability. However, working with this type of robot must imply solving complex and computationally not only inverse/forward kinematics problems but also dynamics ones, which are complicated. Meanwhile, the inverse kinematics issue is tractable by geometrical methods [3,4], but the forward kinematics problem involves a system of nonlinear equations that usually has no closed-form solution. Therefore, the artificial neural networks (ANNs) method is proposed for finding a unique closed-form analytic solution to the forward kinematics problem [5]. There have been few studies on the dynamic modeling of the experimental configuration Hexa parallel robot [6,7]. In-depth study of the Hexa robot dynamics is needed to take full advantage of its strengths for trajectory control and fault detection.

It is a critical issue to study the collisions of a robot manipulator due to diverse application scenarios. An approach for accurate and real-time monitoring of serial robot manipulator collisions is presented in [8], which uses proprioception and IMU sensing for velocity and acceleration estimation. Another observer method to adapt the erroneous dynamics model of the serial manipulators is introduced in [9], where the observer significantly improves the collision detection sensitivity. Sensorless collision detection approaches based on different methods are presented in [10,11,12]. Accelerometers are attached along the surface of the robot arm to detect and localize contact events [13]. Tactile sensors mounted with the robot manipulator are applied to provide a tactile perception of the robot [14,15,16]. In [17,18], a virtual force sensor has been proposed for collision detection and estimating external forces. Authors of [19] have used neural networks for collision detection and classification. Most of the above methods are extremely difficult to apply to parallel manipulators. However, some papers use the dynamics equations and first-order filter structure in combination to build an observer as the collision identification signal called a residual [20]. The residual equals zero up to noise and disturbances during free motion or higher than a suitable threshold in response to a collision. The performance of this method is suitable not only for robot manipulators, and many pieces of research related to humanoid robots have been carried out [21,22].

Recently, a vision-based control system has been widely utilized in robotics to provide information about the target objects or the robot’s pose as position and orientation. Besides that, many vision-based techniques during the isolation phase have been presented. The authors of [23,24] have analyzed the problem of sensor-based collision detection and isolation for a robot manipulator using a set of images taken from several stationary cameras. A system combining deep learning and stereo vision for object detection, classification, and distance calculation [24] can be effectively applied for not only autonomous robot navigation but also collision isolation. For moving object detection, a method of estimating the motion of objects and extracting shapes of detected objects from stereo video cameras has been proposed in [25]. Another system with dual-depth camera information and visual odometry was used to detect moving objects, and an adaptive thresholding method was adopted to enhance performance [26]. An algorithm based on background subtraction and edge detection was applied to detect and segment objects in video frames [27]. In the collision isolation phase, moving objects in the workspace should be detected based on the multi-camera system, using the advantages of image processing techniques. One of them is the interesting You-Only-Look-Once (YOLO) algorithm, firstly presented by Redmon et al. in [23,28], so the contact point location and the Hexa robot pose in this paper are determined with the improved algorithm.

After collision detection and collision isolation, it is essential to determine the suitable method for identifying the contact force. In [29], data from a 6D wrist Force/Torque sensor is combined with the probabilistic approach to link contact estimation based on geometric considerations and compliant motions. In [12,30], the time series model and fuzzy identification were used for determining contact location. Once the contact point is detected, the exchanged force estimation must be the next stage for a complete identification phase. Some dynamics algorithms are approached to perform the external force estimation, such as the recursive Newton–Euler algorithm (RNEA) for the inverse dynamics [31,32], the articulated body algorithm (ABA) for the forward dynamics, and the composite-rigid-body algorithm (CRBA) for calculating the joint-space inertia matrix (JSIM) [33,34]. A recursive Newton-Euler method for inverse dynamics is suitable to analyze external forces acting on the Hexa parallel robot. Based on the above discussion, this paper is motivated by the primary algorithms to solve the detected collision issues as follows:

- The analysis of implementing a dynamic model of the moving Hexa parallel robot for the collision detection problem is proposed.
- The obstacles and the Hexa robot are isolated by the proposed geometrical algorithm and the set of real-time images taken from the multi-cameras system is used to locate the contact point.
- Finally, the Cartesian external force at the contact point is estimated by the recursive Newton-Euler algorithm for the Hexa parallel robot, compared to the force sensor in different collision point cases.

The rest of this paper is organized as follows. We present the configuration, the kinematics and dynamics problems of the Hexa robot in Section 2. In Section 3, the proposed collision detection, isolation, and identification methods applied to the Hexa robot are presented. We show block diagrams to illustrate the entire Hexa robot system and introduce equipment used in this study in Section 4. Section 5 includes the experimental scheme of study cases where the proposed methods are validated based on the given trajectories. Finally, we conclude the paper in Section 6.

## 2. Modeling of the Hexa Parallel Robot

### 2.1. Configurations of the Hexa Robot

**Figure 1:**
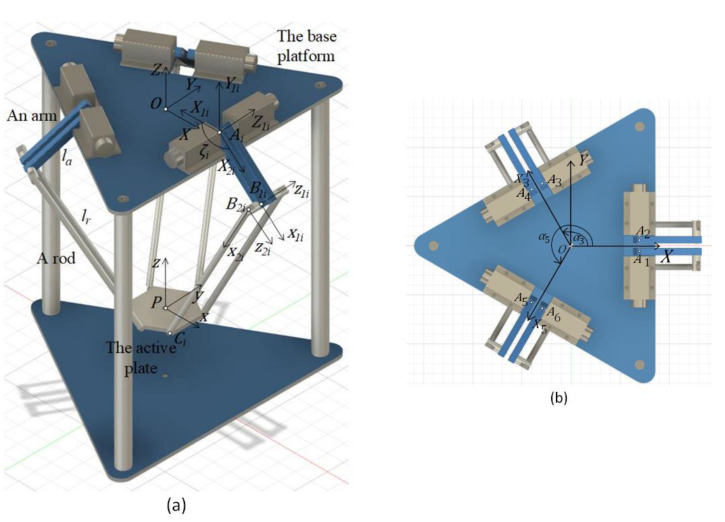
The model of the Hexa robot: (**a**) in 3D view, (**b**) in top view.

The model of the Hexa parallel robot with six serial kinematics chains is shown in Figure 1. Each kinematic chain is described as a revolute-universal-spherical chain link between the base platform and the active plate. The arms driven by six motors at revolute joints connect with the rods at the universal joints. These rods are associated with the active plate at spherical joints. There are two main coordinate frames when the Hexa robot is considered. The first fixed coordinate frame {O}={OXYZ} is located in the center of the base platform with the Z-axis perpendicular to the base platform upwards. The X-axis direction points from the origin O to the midpoint of the edge $A_{1}A_{2}$. The Y-axis is determined by the right-hand rule. The second rotating coordinate frame {P}={Pxyz} is located in the center of the active plate with the z-axis perpendicular to the surface of the active plate. The x-axis direction points from the end-effector to the midpoint of the edge $C_{1}C_{2}$. The y-axis is determined in the same way as as the *Y*-axis. For the ith kinematic chain (i=1,…,6), the point $A_{i}$ represents the center of the revolute joint located in the ith arm. The point $B_{i}$ is the center of the universal joint that connects the ith rod with the ith arm. The point $C_{i}$ is the center of the spherical joint that links between the ith rod and the active plate. The length of the ith arm and the ith rod are $l_{a}$ and $l_{d}$, respectively. The angle $\zeta_{i}$ is the rotation angle of the revolute joint of the ith arm.

The point P representing the end-effector and the Euler angles (ϕ, θ, ψ) of the active plate with respect to the fixed frame {O} are given. Three angles (ϕ, θ, ψ) are defined in compliance with the intrinsic rotation convention of Euler angles. The coordinate with respect to the active frame {P} can be transformed to the coordinate with respect to the fixed frame {O} by using the rotation matrix $R_{P}^{O}$:

(1) $$R_{P}^{O}=\left[\begin{array}{ccc} \cos\phi \cos\theta \cos\psi -\sin\phi \sin\psi & -\cos\phi \cos\theta \sin\psi -\sin\phi \cos\psi & \cos\phi \sin\theta \\ \sin\phi \cos\theta \cos\psi +\cos\phi \sin\psi & -\sin\phi \cos\theta \sin\psi +\cos\phi \cos\psi & \sin\phi \sin\theta \\ -\sin\theta \cos\psi & \sin\theta \sin\psi & \cos\theta \end{array}\right]$$

The position and orientation of the active plate can be determined by three Euler angles and the position of point P:

(2) $$r_{P}^{O}={\left[x_{P}\;y_{P}\;z_{P}\;\right]}^{T}$$

The angular velocity $\omega_{P}$ of the active plate can be expressed as:

(3) $$\omega_{P}=\left[\begin{array}{c} \dot{\psi }\cos\phi \sin\theta -\dot{\theta }\sin\phi \\ \dot{\psi }\sin\phi \sin\theta +\dot{\theta }\cos\phi \\ \dot{\psi }\cos\theta +\dot{\phi } \end{array}\right]$$

At the point O on the base platform, three frames $\left\{O_{i}\right\}=\left\{O_{i}X_{i}Y_{i}Z_{i}\right\}$ (i=1,3,5) are built with the $Z_{i}$-axis coinciding with the Z-axis. The $X_{i}$-axis direction points to the center of edge $A_{i}A_{i+1}$. The $Y_{i}$-axis is determined by the right-hand rule. Simultaneously, at the point P on the active plate, three frames $\left\{P_{i}\right\}=\left\{P_{i}x_{i}y_{i}z_{i}\right\}$ (i=1,3,5) are also constructed. The rotation matrix of frame $\left\{O_{i}\right\}$ relative to frame {O} and frame $\left\{P_{i}\right\}$ relative to frame {P} can be expressed as:

(4) $$R_{O_{i}}^{O}=R_{P_{i}}^{P}=\left[\begin{array}{ccc} \cos\alpha_{i} & -\sin\alpha_{i} & 0\\ \sin\alpha_{i} & \cos\alpha_{i} & 0\\ 0 & 0 & 1 \end{array}\right]$$

where

(5) $${\left[\alpha_{1}\;\alpha_{2}\;\alpha_{3}\;\alpha_{4}\;\alpha_{5}\;\alpha_{6}\right]}^{T}={\left[0\;0\;\frac{2\pi }{3}\;\frac{2\pi }{3}\;\frac{4\pi }{3}\;\frac{4\pi }{3}\right]}^{T}$$

At the point $A_{i}$ on the base platform, a frame $\left\{A_{1i}\right\}=\left\{A_{1i}X_{1i}Y_{1i}Z_{1i}\right\}$ (i=1,…,6) is built with the $Z_{1i}$-axis along with the rotation axis. The $Y_{1i}$-axis is perpendicular to the base platform upwards. The $X_{1i}$-axis is determined by the right-hand rule. The rotation matrix of frame $\left\{A_{1i}\right\}$ relative to frame $\left\{O_{i}\right\}$ is:

(6) $$R_{A_{1i}}^{O_{i}}=\left[\begin{array}{ccc} -1 & 0 & 0\\ 0 & 0 & 1\\ 0 & 1 & 0 \end{array}\right]$$

At the point $A_{i}$, a frame $\left\{A_{2i}\right\}=\left\{A_{2i}X_{2i}Y_{2i}Z_{2i}\right\}$ (i=1,…,6) attached to the ith arm is established with the $Z_{2i}$-axis coinciding with the $Z_{1i}$-axis. The $X_{2i}$-axis coincides with the axial line of the ith arm. The $Y_{2i}$-axis is determined by the right-hand rule. The rotation matrix of frame $\left\{A_{2i}\right\}$ relative to frame $\left\{A_{1i}\right\}$ is:

(7) $$R_{A_{2i}}^{A_{1i}}=\left[\begin{array}{ccc} \cos\zeta_{i} & -\sin\zeta_{i} & 0\\ \sin\zeta_{i} & \cos\zeta_{i} & 0\\ 0 & 0 & 1 \end{array}\right]$$

where $\zeta_{i}$ is the rotation angle of the revolute joint of the arm.

At the point $B_{i}$, a frame $\left\{B_{1i}\right\}=\left\{B_{1i}x_{1i}y_{1i}z_{1i}\right\}$ (i=1,…,6) attached to the ith arm and a frame $\left\{B_{2i}\right\}=\left\{B_{2i}x_{2i}y_{2i}z_{2i}\right\}$ (i=1,…,6) attached to the ith rod are built with the $x_{1i}$-axis pointing from $A_{i}$ to $B_{i}$. The $x_{2i}$-axis points from $B_{i}$ to $C_{i}$. The $z_{1i}$-axis and the $z_{2i}$-axis coincide with the two rotating axes of the universal joint, respectively. The $y_{1i}$-axis and the $y_{2i}$-axis are determined by the right-hand rule. The rotation matrix of frame $\left\{B_{1i}\right\}$ relative to frame $\left\{A_{2i}\right\}$ is:

(8) $$R_{B_{1i}}^{A_{2i}}=\left[\begin{array}{ccc} 1 & 0 & 0\\ 0 & 1 & 0\\ 0 & 0 & 1 \end{array}\right]$$

The rotation matrix of the frame $\left\{B_{2i}\right\}$ relative to the frame $\left\{B_{1i}\right\}$ can be calculated by transformations. Initially, the points of $A_{i}$, $B_{i}$, and $C_{i}$ align on the same line. The frame $\left\{B_{2i}\right\}$ is obtained by rotating the frame $\left\{B_{1i}\right\}$ around the two rotating axes of the universal joint by the two rotation angles $\beta_{i}$ and $\gamma_{i}$, respectively. The rotation matrix of frame $\left\{B_{2i}\right\}$ relative to frame $\left\{B_{1i}\right\}$ is:

(9) $$R_{B_{2i}}^{B_{1i}}=\left[\begin{array}{ccc} \cos\beta_{i}\cos\gamma_{i} & -\cos\beta_{i}\sin\gamma_{i} & \sin\beta_{i}\\ \sin\beta_{i}\cos\gamma_{i} & -\sin\beta_{i}\sin\gamma_{i} & -\cos\beta_{i}\\ \sin\gamma_{i} & \cos\gamma_{i} & 0 \end{array}\right]$$

Finally, the rotation matrix of the frame $\left\{A_{2i}\right\}$ relative to the frame {O} and the frame $\left\{B_{2i}\right\}$ relative to the frame {O} are:

(10) $$R_{A_{2i}}^{O}=R_{O_{i}}^{O}R_{A_{1i}}^{O_{i}}R_{A_{2i}}^{A_{1i}}=\left[\begin{array}{ccc} -\cos\alpha_{i}\cos\zeta_{i} & \cos\alpha_{i}\sin\zeta_{i} & -\sin\alpha_{i}\\ -\sin\alpha_{i}\cos\zeta_{i} & \sin\alpha_{i}\sin\zeta_{i} & \cos\alpha_{i}\\ \sin\zeta_{i} & \cos\zeta_{i} & 0 \end{array}\right]$$

(11) $$R_{B_{2i}}^{O}=R_{O_{i}}^{O}R_{A_{1i}}^{O_{i}}R_{A_{2i}}^{A_{1i}}R_{B_{1i}}^{A_{2i}}R_{B_{2i}}^{B_{1i}}=\left[\begin{array}{ccc} R_{11} & R_{12} & R_{13}\\ R_{21} & R_{22} & R_{23}\\ R_{31} & R_{32} & R_{33} \end{array}\right]$$

where

(12) $$\beta_{i}=-\zeta_{i}-\arctan\left(\frac{R_{31}}{R_{11}\cos\alpha_{i}+R_{21}\sin\alpha_{i}\;}\right)\;\mathrm{with}-\frac{\pi }{2}<\beta_{i}+\zeta_{i}<\frac{\pi }{2}\;\gamma_{i}=\arcsin\left(R_{21}\cos\alpha_{i}-R_{11}\sin\alpha_{i}\right)\;\mathrm{with}-\frac{\pi }{2}<\gamma_{i}<\frac{\pi }{2}$$

and

(13) $$R_{11}=-\sin\alpha_{i}\sin\gamma_{i}-\cos\alpha_{i}\cos\left(\beta_{i}+\zeta_{i}\right)\cos\gamma_{i}\;R_{12}=-\sin\alpha_{i}\cos\gamma_{i}+\cos\alpha_{i}\cos\left(\beta_{i}+\zeta_{i}\right)\sin\gamma_{i}\;R_{13}=-\cos\alpha_{i}\sin\left(\beta_{i}+\zeta_{i}\right)\;R_{21}=\cos\alpha_{i}\sin\gamma_{i}-\sin\alpha_{i}\cos\left(\beta_{i}+\zeta_{i}\right)\cos\gamma_{i}\;R_{22}=\cos\alpha_{i}\cos\gamma_{i}+\sin\alpha_{i}\cos\left(\beta_{i}+\zeta_{i}\right)\sin\gamma_{i}\;R_{23}=-\sin\alpha_{i}\sin\left(\beta_{i}+\zeta_{i}\right)\;R_{31}=\sin\left(\beta_{i}+\zeta_{i}\right)\cos\left(\gamma_{i}\right)\;R_{32}=-\sin\left(\beta_{i}+\zeta_{i}\right)\sin\left(\gamma_{i}\right)\;R_{33}=-\cos\left(\beta_{i}+\zeta_{i}\right)$$

### 2.2. Kinematics

#### 2.2.1. Inverse Kinematics

**Figure 2:**
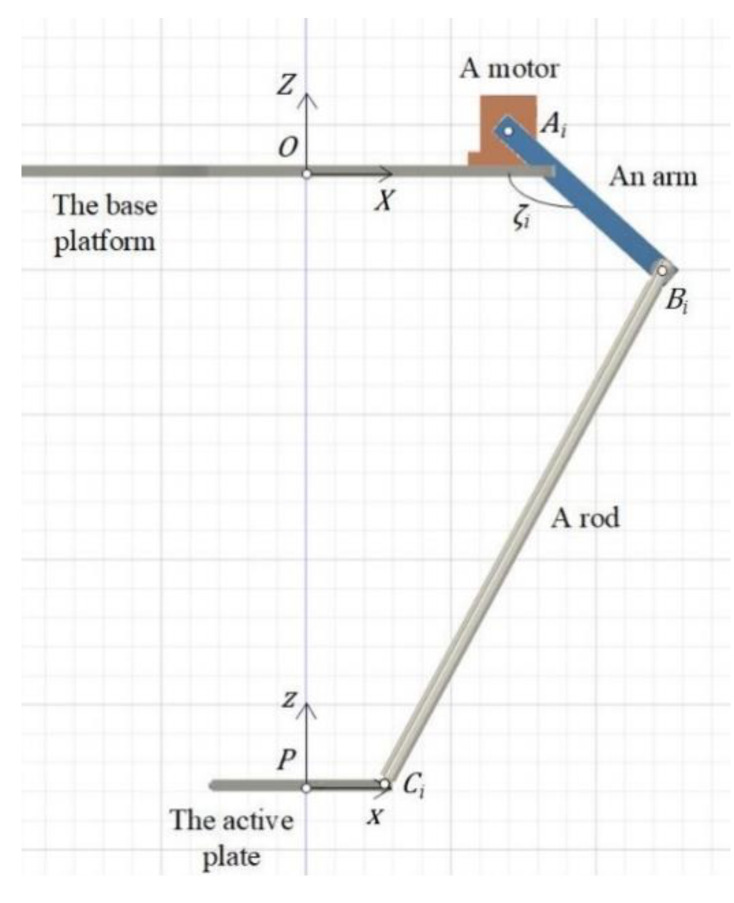
A kinematics chain of the Hexa robot.

In the inverse kinematics, the position and Euler angles of the active plate are given. The rotation angles of motors need to be determined. A kinematics chain of the Hexa robot is shown in Figure 2. The coordinate of point $A_{i}$ in frame {O} and $C_{i}$ in frame {P} are given. The vector $r_{C_{i}}^{O}$ can be expressed as follows:

(14) $$r_{C_{i}}^{O}=r_{A_{i}}^{O}+AB_{i}+BC_{i}=r_{P}^{O}+PC_{i}$$

The kinematics equations can be expressed as follows:

(15) $$\begin{array}{l} l_{r}^{2}=l_{a}^{2}+{\left(x_{A_{i}}-x_{C_{i}}\right)}^{2}+{\left(y_{A_{i}}-y_{C_{i}}\right)}^{2}+{\left(z_{A_{i}}-z_{C_{i}}\right)}^{2}\\ \ldots \ldots +\left(-2l_{a}\cos\alpha_{i}\left(x_{A_{i}}-x_{C_{i}}\right)-2l_{a}\sin\alpha_{i}\left(y_{A_{i}}-y_{C_{i}}\right)\right)\cos\zeta_{i}+(2l_{a}(z_{A_{i}}\\ -z_{C_{i}}))\sin\zeta_{i} \end{array}$$

where

(16) $$r_{C_{i}}^{O}={\left[x_{C_{i}}\;y_{C_{i}}\;z_{C_{i}}\;\right]}^{T}\;r_{A_{i}}^{O}={\left[x_{A_{i}}\;y_{A_{i}}\;z_{A_{i}}\;\right]}^{T}$$

(17) $$a_{i}=l_{r}^{2}-l_{a}^{2}-{\left(x_{A_{i}}-x_{C_{i}}\right)}^{2}-{\left(y_{A_{i}}-y_{C_{i}}\right)}^{2}-{\left(z_{A_{i}}-z_{C_{i}}\right)}^{2}\;b_{i}=-2l_{a}\cos\alpha_{i}\left(x_{A_{i}}-x_{C_{i}}\right)-2l_{a}\sin\alpha_{i}\left(y_{A_{i}}-y_{C_{i}}\right)\;c_{i}=2l_{a}\left(z_{A_{i}}-z_{C_{i}}\right)\;\mathrm{with}\;i=1,\ldots ,\;6$$

The rotation angle $\zeta_{i}$ can be calculated as the following expression:

(18) $$\zeta_{i}=\arctan\frac{a_{i}c_{i}^{2}-b_{i}\sqrt{-c_{i}^{2}\left(a_{i}^{2}-b_{i}^{2}-c_{i}^{2}\right)}}{c_{i}\left(a_{i}b_{i}+\sqrt{-c_{i}^{2}\left(a_{i}^{2}-b_{i}^{2}-c_{i}^{2}\right)}\right)}$$

#### 2.2.2. Forward Kinematics

**Figure 3:**
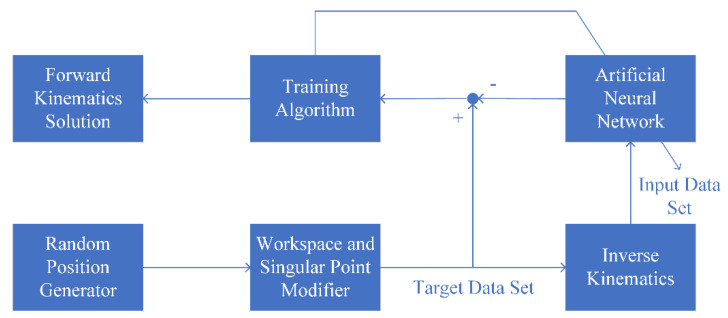
The procedure of neural network training [3].

The procedure of neural network training is presented in Figure 3. In the forward kinematics problem, the rotation angles of the arms are given, and the pose of the end-effector is determined by solving kinematics equations. These equations are nonlinear, so analytically, finding their solutions is highly complicated. For the above reason, artificial neural networks (ANNs) are employed to calculate the approximate pose of the end-effector. Firstly, a data set consisting of the potential pose positions of the active plate is created randomly. This data set should be modified to reject the singular points and points outside the workspace. The modified data set is called the target data in ANN training. From the target data, the inverse kinematics problem is solved and the data set of angles $\zeta_{i}$ of six arms is created. This data set is called the input data in ANN training. The training results for weight and bias factors are used to calculate the forward kinematics problem.

### 2.3. Dynamics

The dynamics equations can be obtained by several methods, such as the Newton-Euler Formulation, Lagrange equations of the first type, and the Virtual Work Principle. In this study, the Lagrange equations of the first type are applied to derive dynamics equations. Those equations are arranged into two sets and expressed as follows:

(19) $$\frac{d}{dt}\left(\frac{\partial L}{\partial {\dot{q}}_{j}}\right)-\frac{\partial L}{\partial q_{j}}=\tau_{j}+\overset{6}{\underset{i=1}{\sum }}\lambda_{i}\frac{\partial f_{i}}{\partial q_{j}}\;\;\left(j=1,\ldots ,\;6\right)$$

(20) $$\frac{d}{dt}\left(\frac{\partial L}{\partial {\dot{q}}_{j}}\right)-\frac{\partial L}{\partial q_{j}}=Q_{j}+\overset{6}{\underset{i=1}{\sum }}\lambda_{i}\frac{\partial f_{i}}{\partial q_{j}}\;\;\left(j=7,\ldots ,\;12\right)$$

where $q={\left[\zeta_{1}\;\zeta_{2}\;\zeta_{3}\;\zeta_{4}\;\zeta_{5}\;\zeta_{6}\;x_{p}\;y_{p}\;z_{p}\;\phi \;\theta \;\psi \right]}^{T}$ presents the generalized coordinates; L denotes the Lagrangian function of the whole system, which is the Lagrangian functions summation of the active plate, the arms, and the rods; $\tau_{j}=K_{t}I_{j}$ is the actuator torque; $K_{t}$ is the motor torque constant and $I_{j}$ is the motor armature current; $Q_{j}$ is the generalized force; $\lambda_{i}$ (i=1,…,6) is the ith Lagrangian multiplier; $f_{i}=\left(b_{i}\cos\zeta_{i}+c_{i}\sin\zeta_{i}-a_{i}\right)$ is the ith constraint equation; $a_{i}$, $b_{i}$, and $c_{i}$ are in Equation (17).

#### 2.3.1. Inverse Dynamics

Twelve motion variables $q_{j}$ and six generalized forces $\tau_{j}$ contributed by the actuators are given. Solving the inverse dynamics problem is to find out the six generalized external forces.

The first six-equation set Equation (19) contains the six Lagrange multipliers $\lambda_{i}$ (i=1,…,6) as the unknowns. The first set can be written in the form:

(21) $$\overset{6}{\underset{i=1}{\sum }}\lambda_{i}\frac{\partial f_{i}}{\partial q_{j}}=\frac{d}{dt}\left(\frac{\partial L}{\partial {\dot{q}}_{j}}\right)-\frac{\partial L}{\partial q_{j}}-\tau_{j}\;\left(j=1,\ldots ,\;6\right)$$

The six generalized external forces $Q_{j}$ (j=7,…,12) can be calculated by solving the second six-equation set Equation (20):

(22) $$Q_{j}=\frac{d}{dt}\left(\frac{\partial L}{\partial {\dot{q}}_{j}}\right)-\frac{\partial L}{\partial q_{j}}-\overset{6}{\underset{i=1}{\sum }}\lambda_{i}\frac{\partial f_{i}}{\partial q_{j}}\left(j=7,\ldots ,\;12\right)$$

#### 2.3.2. Forward Dynamics

Six generalized external forces $Q_{j}$ and six generalized forces $\tau_{j}$ contributed by the actuators are given. Solving the forward dynamics problem is to find out the twelve motion variables $q_{j}$.

Analyzing the problem, we have eighteen equations, including twelve dynamics equations in Equations (19) and (20), and six kinematics equations in Equation (15). There are eighteen unknown variables, including twelve motion variables $q_{j}$ and six Lagrange multipliers $\lambda_{i}$ (i=1,…,6). This equation system is a differential-algebraic equations system containing differential equations and algebraic equations. The strategy to solve this system of equations is to reduce differential order, leading to an increment in the number of variables and equations. Then, this system of equations becomes algebraic equations. MATLAB has a function to solve differential algebraic equations (DAEs), but solving forward dynamics problems is still extremely complicated.

## 3. Collision Detection, Isolation and Identification of the Hexa Robot

### 3.1. Collision Detection

When robot manipulators move freely in the environment, an obstacle may come to the workspace of the robot manipulator at any time. If that obstacle collides with the robot, a collision has occurred. Implementing collision detection is to recognize whether a robot collision occurred or not. An observer calculated based on the dynamics equations can detect the occurrence of a collision. A suitable threshold is selected based on the requirement of the accuracy for this observer. A collision occurs if the observer signal is greater than the chosen threshold.

The generalized momentum of the Hexa robot is defined as:

(23) $$p_{j}=\frac{\partial L}{\partial {\dot{q}}_{j}}\left(j=1,\ldots ,12\right)$$

According to the second dynamics equations set Equation (19), the generalized force can be written in the form:

(24) $$Q_{j}={\dot{p}}_{j}-\frac{\partial L}{\partial q_{j}}-\overset{6}{\underset{i=1}{\sum }}\lambda_{i}\frac{\partial f_{i}}{\partial q_{j}}\left(j=7,\ldots ,\;12\right)$$

The collision identification signal, or simply the residual r, is:

(25) $$\dot{r}=-Kr+KQ$$

Substituting $Q_{j}$ from (24) into (25), we obtain:

(26) $${\dot{r}}_{j}=-K_{j}r_{j}+K_{j}\left({\dot{p}}_{j}-\frac{\partial L}{\partial q_{j}}-\overset{6}{\underset{i=1}{\sum }}\lambda_{i}\frac{\partial f_{i}}{\partial q_{j}}\right)\;\left(j=7,\ldots ,12\right)$$

Lagrange multipliers $\lambda_{i}$ (i=1,…,6) can be computed using the first dynamics equations set Equation (19). When a collision occurs, at least an element $r_{j}$ of vector r raises with a time constant 1/$K_{j}$. The detection is performed as soon as $\left|r_{j}\right|>r_{low}$, where $r_{low}\;$is the chosen threshold.

### 3.2. Collision Isolation

**Figure 4:**
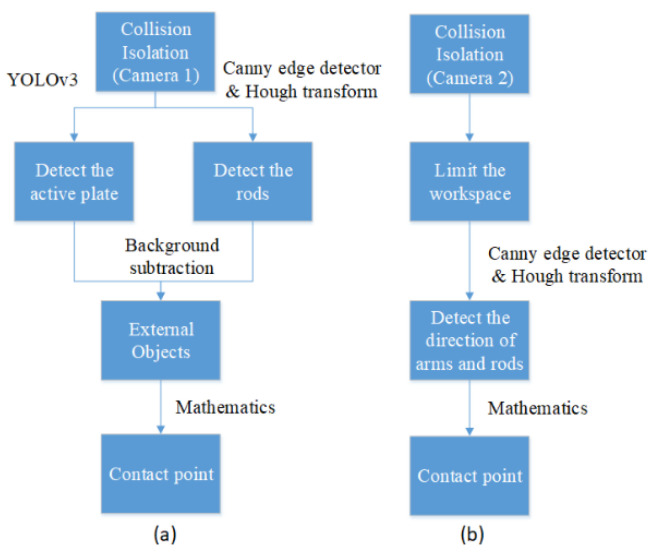
The flowchart of the collision isolation procedure: for the first camera (**a**); for the second camera (**b**).

**Figure 5:**
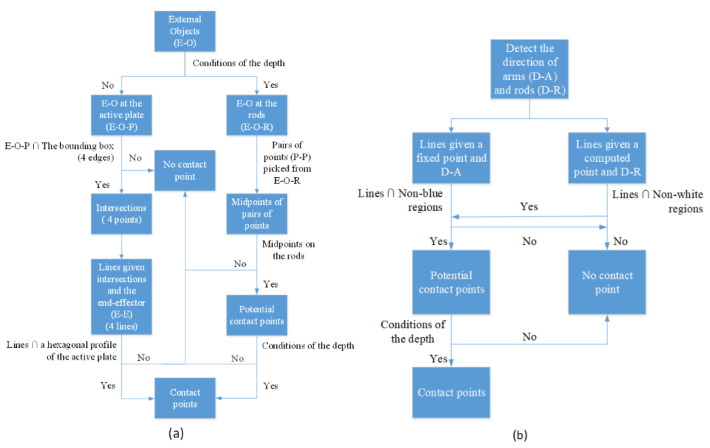
The flowchart of the mathematics processing for the first camera (**a**); the second camera (**b**).

After the residual indicates that a collision occurs on the Hexa robot, it is necessary to identify the collision location. Collision isolation aims at localizing the contact point. In our paper, a vision system consisting of dual depth cameras is applied to detect the contact point if a collision occurs. The first camera’s photos at the time of a collision case are analyzed to identify whether the contact point is at the active plate or the rods. Otherwise, the contact point is detected by processing the image captured by the second camera. Based on the multi-camera vision system, we propose the flowchart of the collision isolation procedure in Figure 4 and the mathematics processing for localization of the contact point in Figure 5.

In this study, every external object coming to the workspace should be from the outside and cause the Hexa robot obscuration. Because the whole Hexa parallel manipulator cannot be observed in only images captured by the first dual depth camera, this camera is responsible for the external object isolation on the active plate and the rods from these images. On the contrary, the external obstacle for the arms case is identified by the second dual depth camera. Both arm and rod situations are considered in the direction to see the obstacle obscuring them.

- Case 1: Contact points are determined by the first camera.

Firstly, a bounding box around the active plate is recognized using YOLOv3. Because the color of the rods is different from the background, the rods can be isolated from the image, so the RGB color image is converted to a YCbCr color image. Then, the Canny edge detector and Hough transform are applied to recognize straight-line profiles around rods. From these detected lines, the rods are identified in the image. The considered image and the background image are converted to grayscale images. A binary image is created by subtracting the grayscale background image from the grayscale considered image. After combining the image subtraction with depth conditions of the objects in the workspace, the external object is identified by removing the Hexa robot from the binary image.

To find the contact point on the active plate, all points belonging to the external object are checked to see if any of them touch the bounding box around the active plate. Then, the image depth is considered at these points. The difference between the depth of these points and the bounding box must be less than a chosen threshold. A point represents intersections of the external object and the bounding box around the active plate, which is obtained by taking an average. A line passes through the mentioned point and the end-effector. Finally, the contact point is located at the junction of the line and the active plate’s hexagonal border.

To find the contact point on a rod, the possible pairs of points belonging to the external object are checked respectively to see if the midpoint of these points is on the rod. Then, the image depth is considered at these points. The difference between these points’ depth and the midpoint’s depth on this rod must be less than the chosen threshold. All of these midpoints are probably the contact point. As a result, a point represents the contact point location, obtained by averaging all the midpoint locations.

- Case 2: Contact points are determined by the second camera.

Based on the image depth, only objects in a limited workspace are considered in the image captured from the second camera. Because the color of the arms is blue, the arms can be isolated from the image. An appropriate threshold for the HSV color scale is chosen for probable extracting of the blue region from the image. Then, the Canny edge detector and Hough transform are applied to recognize straight-line profiles along arms. The direction of arms can be identified from these lines. Two fixed points are chosen as the motor location where two arms are connected with two motors. According to the fixed points and the directions of the arms, all points belonging to the blue region in the arm directions are checked to see if any of them intersect the non-blue region. The intersections are probable contact point locations. The correct contact point location can be detected with the depth conditions.

After the RGB color image is converted to a YCbCr color image, the rods can be isolated from the image. The Canny edge detector and Hough transform are applied to recognize straight-line profiles along rods. The direction of rods is calculated from these profiles. Two points are computed as the connector location where two arms are connected with two rods. According to the computed points and the direction of the rods, the white region points in the rod direction are checked for the non-white intersect probability. The intersections are probable contact point locations. The correct location of the contact point is determined based on the depth conditions and the above junctions.

### 3.3. Collision Identification

After the collision occurs and the contact point location is known, the information on the contact force should be determined. This study applies a Recursive Newton-Euler Algorithm (RNEA) to calculate how much the contact force was instead of using more devices. The inputs for this algorithm are the current intensity measured from current sensors and the pose of the end-effector obtained from image processing.

Three cases correspond to three possibilities that a collision occurs at an arm, a rod, or the active plate. RNEA is developed separately for the application with the Hexa robot in each case.

(27) $$F_{ai}=m_{a}{\dot{v}}_{ai}\;M_{ai}=I_{ai}{\dot{\omega }}_{ai}+\omega_{ai}\times \left(I_{ai}\omega_{ai}\right)\;F_{ri}=m_{r}{\dot{v}}_{ri}\;M_{ri}=I_{ri}{\dot{\omega }}_{ri}+\omega_{ri}\times \left(I_{ri}\omega_{ri}\right)\;F_{p}=m_{p}{\dot{v}}_{p}\;M_{p}=I_{p}{\dot{\omega }}_{p}+\omega_{p}\times \left(I_{p}\omega_{p}\right)$$

where $F_{ai}$ is the inertia force acting on the ith arm; $M_{ai}\;$is the inertia moment acting on the ith arm; $m_{a}$ is the mass of the arm; $v_{ai}$ is the linear velocity of the center of mass (CoM) of the ith arm; $I_{ai}$ is the tensor of mass moment of inertia of the ith arm; $\omega_{ai}$ is the angular velocity of the ith arm; $F_{ri}$ is the inertia force acting on the ith rod; $M_{ri}$ is the inertia moment acting on ith rod; $m_{r}$ is the mass of the rod; $v_{ri}$ is the linear velocity of the center of mass (CoM) of the ith rod; $I_{ri}$ is the tensor of mass moment of inertia of the ith rod; $\omega_{ri}$ is the angular velocity of the ith rod; $F_{p}$ is the inertia force acting on the active plate; $M_{p}$ is the inertia moment acting on the active plate; $m_{p}$ is the mass of the active plate; $v_{p}$ is the linear velocity of the center of mass (CoM) of the active plate; $I_{p}$ is the tensor of mass moment of inertia of the active plate; $\omega_{p}$ is the angular velocity of the active plate.

- Case 1: A collision occurs on the active plate.

First step: According to six kinematics chains, calculate $f_{B_{i}}$ and $f_{C_{i}}$ (i=1,…,6).

(28) $$AB_{i}\times f_{B_{i}}=n_{A_{i}}-n_{B_{i}}-\frac{1}{2}AB_{i}\times F_{ai}-M_{ai}$$

(29) $$f_{B_{i}}-f_{C_{i}}=F_{ri}\;$$

(30) $$BC_{i}\times f_{C_{i}}=n_{B_{i}}-n_{C_{i}}-\frac{1}{2}BC_{i}\times F_{ri}-M_{ri}$$

where $f_{A_{i}}$ and $n_{A_{i}}$ are the reaction force and the reaction moment exerted on the *i*th arm, a quantity of $n_{A_{i}}$ can be computed by the *i*th motor torque; $f_{B_{i}}$ and $n_{B_{i}}=0$ are the force and the moment exerted on the rod by the arm; $f_{C_{i}}$ and $n_{C_{i}}=0\;$are the force and the moment exerted on the active plate by the *i*th rod.

Second step: Calculate the external force $F_{ep}$.

(31) $$F_{ep}=-\overset{6}{\underset{i=1}{\sum }}f_{C_{i}}+F_{p}$$

- Case 2: A collision occurs at an arm.

First step: Assume collision occurred on the first kinematics chain. According to the five remaining kinematics chains, calculate $f_{B_{i}}$ and $f_{C_{i}}$ (i=2,…,6) according to Equations (28)–(30).

Second step: Calculate $f_{C_{1}}$ based on the force balance equation at the active plate.

(32) $$f_{C_{1}}=-\overset{6}{\underset{i=2}{\sum }}f_{C_{i}}+F_{p}$$

Third step: Calculate the external force $F_{e1}$ on the first arm.

(33) $$AD_{1}\times F_{e1}=n_{B_{1}}-n_{A_{1}}+AB_{1}\times f_{B_{1}}+\frac{1}{2}AB_{1}\times F_{a1}+M_{a1}$$

where D is the contact point, which is determined in the collision isolation.

- Case 3: A collision occurs at a rod.

First step: Assume collision occurred in the first kinematics chain. According to the five remaining kinematics chains, calculate $f_{B_{i}}$ and $f_{C_{i}}$ (i=2,…,6) according to Equations (28)–(30).

Second step: Calculate $f_{C_{1}}$ based on the force balance equation at the active plate according to Equation (32).

Third step: Calculate the external force $F_{e1}$ on the first rod.

(34) $$AB_{1}\times f_{B_{1}}=n_{A_{1}}-n_{B_{1}}-\frac{1}{2}AB_{1}\times F_{a1}-M_{a1}$$

(35) $$f_{B_{1}}+F_{e1}=f_{C_{1}}+F_{r1}$$

(36) $$BD_{1}\times F_{e1}=n_{C_{1}}-n_{B_{1}}+BC_{1}\times f_{C_{1}}+\frac{1}{2}BC_{1}\times F_{r1}+M_{r1}$$

where D is the contact point, which is determined in the collision isolation.

## 4. Experimental Setup

**Figure 6:**
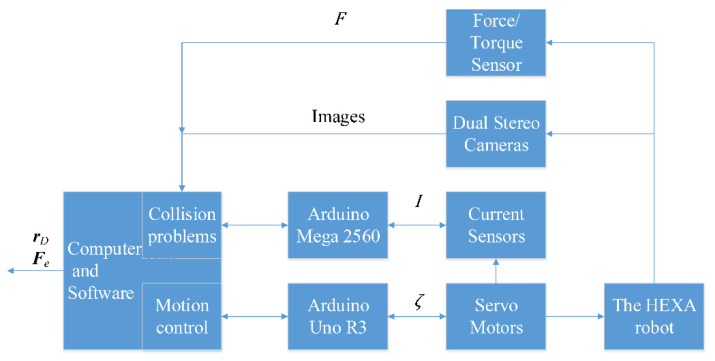
The block diagram of the Hexa robot system.

Figure 6 is the block diagram of the Hexa robot system. Both the computer and control software are responsible for controlling the robot via RS232 and solving collision problems. The robot controller is an Arduino Uno R3 microcontroller board. The six Servo motors are used to drive the arms. A current sensor module measures and sends current signals to Arduino Mega 2560. The vision system, including dual stereo cameras, monitors the Hexa robot. Meanwhile, the computer and software perform image processing. The first camera is mounted under the base platform, and the second camera is fixed away from the workspace of the Hexa robot. A force sensor should be used to verify the accuracy of the algorithm. A force/torque sensor is attached to the active plate to measure forces and torques.

**Figure 7:**
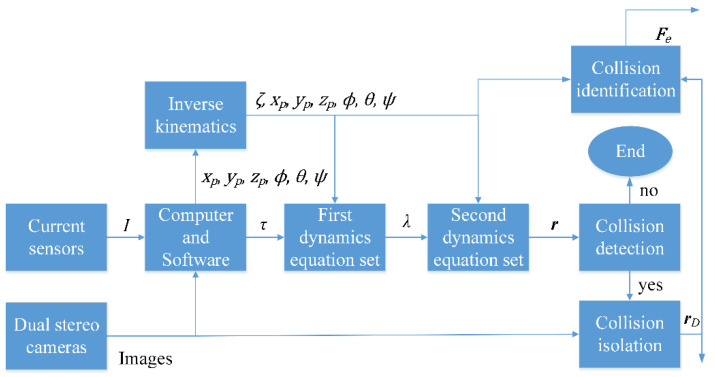
The block diagram of the collision problems solving process.

Figure 7 is the block diagram of the collision problems solving process. In the first step, the end-effector pose ${\left[x_{p}\;y_{p}\;z_{p}\;\phi \;\theta \;\psi \right]}^{T}$ can be obtained from processed images captured by the first camera. Six rotation angles ${\left[\zeta_{1}\;\zeta_{2}\;\zeta_{3}\;\zeta_{4}\;\zeta_{5}\;\zeta_{6}\;\right]}^{T}$ and the actuator torque τ can be sequentially computed by solving the inverse kinematics problems based on the current ${\left[I_{1}\;I_{2}\;I_{3}\;I_{4}\;I_{5}\;I_{6}\;\right]}^{T}$ measured by current sensors. In the second step, with the above variables, Lagrangian multipliers $\lambda_{i}$ (i=1,…,6) can be calculated using the first dynamics equations set. In the third step, the residual $r={\left[r_{1}\;r_{2}\;r_{3}\;r_{4}\;r_{5}\;r_{6}\;\right]}^{T}$ can be computed with the end effector pose and Lagrangian multipliers. According to the residual r, it denotes whether a robot collision occurred or not. In the fourth step, the contact point location $r_{D}$ is determined based on images captured by two stereo cameras. In the final step, after collision isolation, the Recursive Newton-Euler Algorithm is used to identify the external force $F_{e}$. This system is like a sensor wherein the inputs are measured current and the pose of the end-effector, and the outputs are the external contact force and the contact point position. Further research will probably cover exploiting those outputs and regenerating the trajectory.

**Figure 8:**
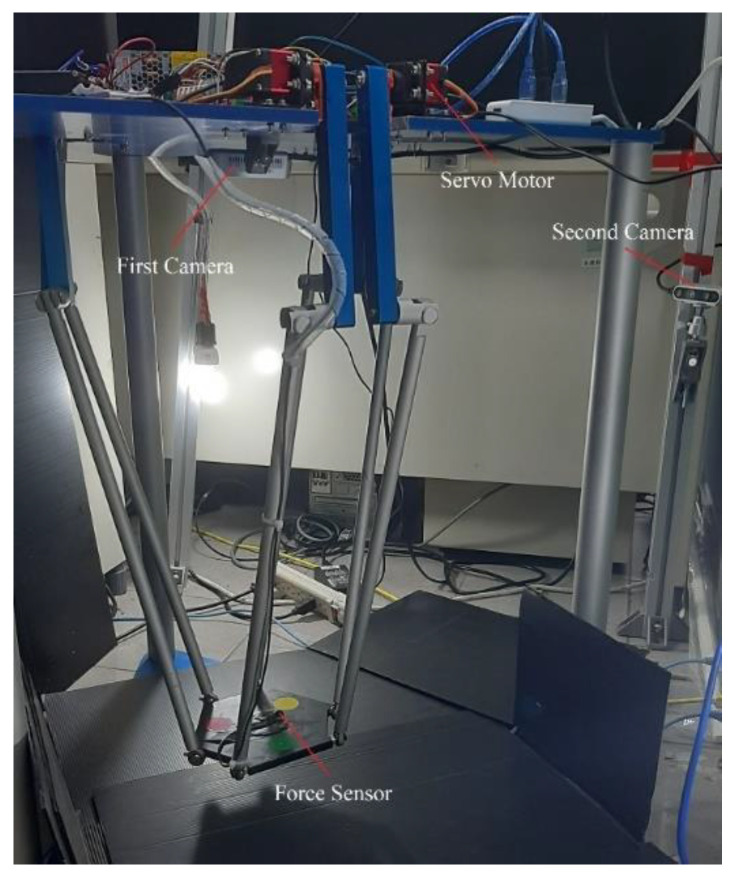
The Hexa robot system.

Figure 8 shows the Hexa robot system used in this study. Six arms are driven by six Servo motors fixed on the base platform. The first stereo camera is mounted under the base platform to monitor the workspace from above. The second stereo camera is fixed on an immovable bar to observe from the outside. The force/torque sensor is mounted on the active plate to evaluate the external force in the experiment.

## 5. Experimental Results

### 5.1. Case 1: Collision at the Active Plate

#### 5.1.1. Collision Detection at the Active Plate

**Figure 9:**
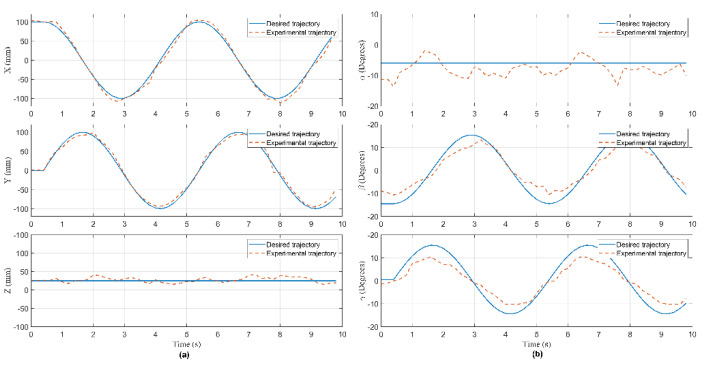
The pose of the end-effector in Case 1: (**a**) The position of the end- effector; (**b**) the orientation of the active plate.

**Figure 10:**
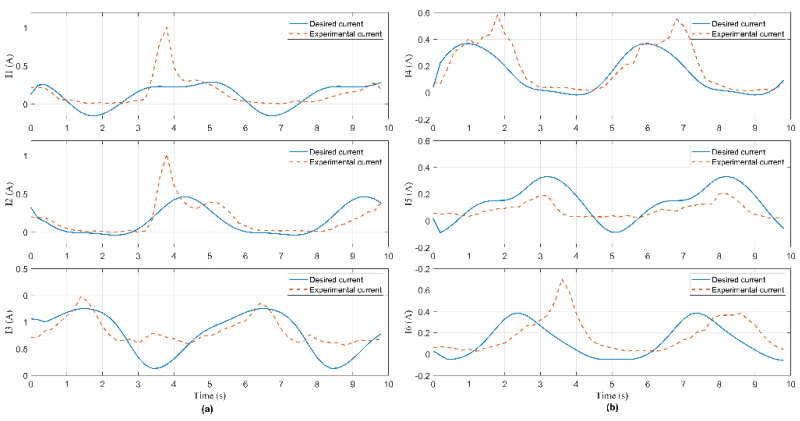
Current signal measured from current sensors in Case 1: (**a**) The 1st, 2nd, and 3rd sensor; (**b**) the 4th, 5th, and 6th sensor.

In this experiment, the Hexa robot is controlled to move the active plate in a circular trajectory in the workspace. A collision occurred due to an external force acting on the active plate. When the active plate moves in a circle trajectory, the first camera mounted under the base platform captures images. These images are processed to compute the pose of the end-effector, shown in Figure 9. The analog voltage signal from six current sensors is continuously sent to the computer via RS232. The measured current intensity is shown in Figure 10. Equations of the circular trajectory are presented as follows:

(37) $$\{\begin{array}{cc} x=100\cos\left(0.4\pi t\right)\;\;\;\;\; & \left(\mathrm{mm}\right)\\ y=100\sin\left(0.4\pi t\right)\;\;\;\;\; & \left(\mathrm{mm}\right)\\ z=-550 & \left(\mathrm{mm}\right)\\ \alpha =-6 & \left(\deg\right)\\ \beta =1-30\cos\left(0.4\pi t\right)\;\; & \left(\deg\right)\\ \gamma =1+30\sin\left(0.4\pi t\right)\;\;\; & \left(\deg\right) \end{array}$$

Three angles (α, β, γ) are defined in compliance with the Z-Y-X extrinsic rotation convention of Tait–Bryan angles. The three angles (α, β, γ) can be converted to the three angles (ϕ, θ, ψ). The rotation matrix is expressed as follows:

(38) $$R=\left[\begin{array}{ccc} \cos\alpha \cos\beta & \cos\alpha \sin\beta \sin\gamma -\sin\alpha \cos\gamma & \cos\alpha \sin\beta \cos\gamma +\sin\alpha \sin\gamma \\ \sin\alpha \cos\beta & \sin\alpha \sin\beta \sin\gamma +\cos\alpha \cos\gamma & \sin\alpha \sin\beta \cos\gamma +\cos\alpha \sin\gamma \\ -\sin\beta & \cos\beta \sin\gamma & \cos\beta \cos\gamma \end{array}\right]$$

**Figure 11:**
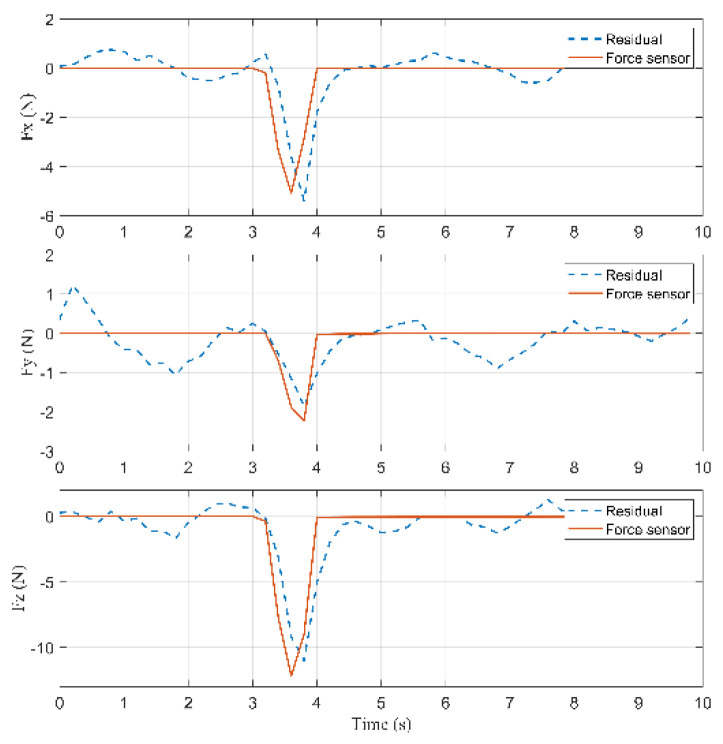
Comparison of the residuals for the force sensor in Case 1.

According to Equation (26), the residuals are computed to detect a collision, shown in Figure 11. Because there is a model error less than 2 (N), a suitable threshold $r_{low}$ is chosen equal to 2 (N). When a collision occurs, the residual $r_{j}$ (j=7,8,9) raises with a time constant 1/K. Detection is performed as soon as $\left|r_{j}\right|>r_{low}$, where $r_{low}$ is the chosen threshold. According to Figure 11, a collision occurred at 3.4 s because the residual in the Z-axis is greater than $r_{low}=2$ (N). The external force reached the maximum value at 3.8 s during the collision. When contact is lost, $\left|r_{j}\right|$ decreases quickly until $\left|r_{j}\right|<r_{low}$.

#### 5.1.2. Collision Isolation at the Active Plate

**Figure 12:**
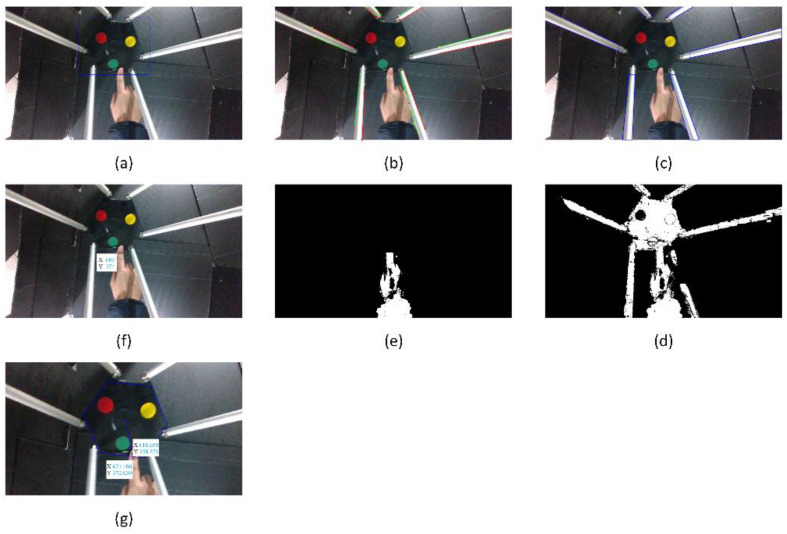
The collision isolation procedure in Case 1: (**a**) The bounding box around the active plate; (**b**) straight lines around rods; (**c**) recognized rods; (**d**) objects in the workspace; (**e**) the external object; (**f**) intersections of the external object and the bounding box; (**g**) the contact point.

The image captured when a collision started occurring is considered to find out the contact point location. According to the flowchart of the procedure of collision isolation shown in Figure 4, a bounding box around the active plate is recognized using YOLOv3, shown in Figure 12. The RGB color image shown above is converted to a YCbCr color image. Then, the Canny edge detector and Hough transform are applied to recognize straight-line profiles around rods. These straight lines are shown in Figure 12b. The rods can be identified in the image, shown in Figure 12c. The considered image and the background image are converted to grayscale images. A binary image is created by subtracting the grayscale background image from the grayscale considered image. After performing image subtraction, objects that are not in the workspace cannot be completely eliminated. Combined with the image depth, only objects in the workspace are considered in the image, which is shown in Figure 12d. Because the Hexa robot, including the active plate and the rods, is determined, the external object can be identified by removing the Hexa robot from the binary image. The image containing the external object is shown in Figure 12e. According to the flowchart of the mathematics processing shown in Figure 5, 180 points are determined to satisfy the conditions. All of them are intersections between the external object and the bounding box around the active plate, shown in Figure 12f. A point represents the above 180 pixels by taking an average. A line passes through the mentioned pixel and the end-effector. According to the marker’s location, the hexagonal profile of the active plate can be calculated. The contact point location is the intersection between the mentioned line and the hexagonal profile, shown in Figure 12g. Following the strategy mentioned above, the contact point location in the considered image is determined as ${\left[358.87\;618.66\right]}^{T}\;$(pixels). According to the camera model, the contact point coordinate is also identified as ${\left[-10.65-48.46-578.45\right]}^{T}$ (mm) when a collision occurs at the active plate.

A collision occurred on the active plate, and the external force reached the maximum value at 3.8 s. Similarly, 155 points are intersections between the external object and the bounding box around the active plate. The contact point location in the considered image is determined as ${\left[406.37\;580.09\right]}^{T}$ (pixels). The contact point coordinate is also identified as ${\left[15.96-71.62-579.72\right]}^{T}$ (mm) when the external force reaches the maximum value.

#### 5.1.3. Collision Identification at the Active Plate

**Figure 13:**
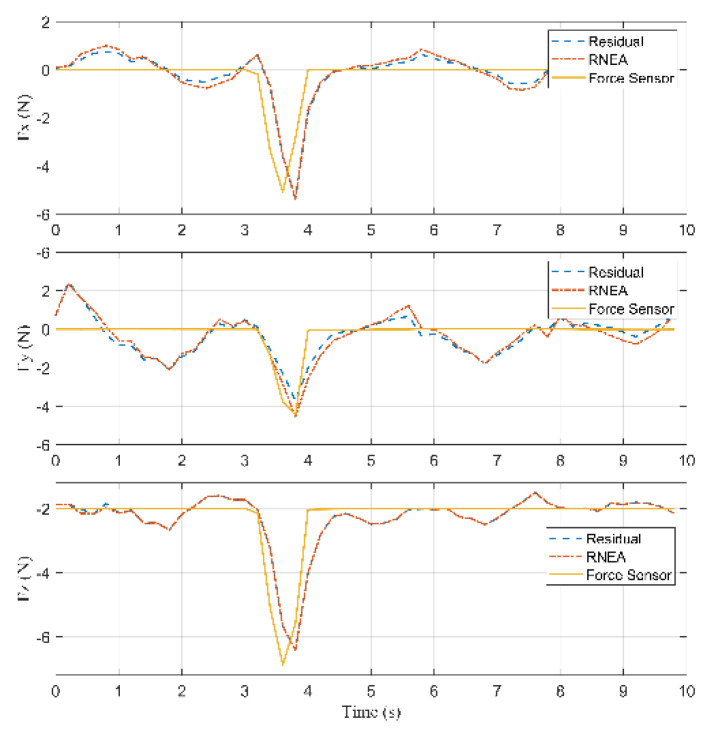
Residuals, the computed forces by RNEA and the force sensor in Case 1.

According to Equation (27), the inertia forces acting on the arms, the rods, and the active plate are calculated. According to Equations (28)–(31), the external force acting on the active plate can be calculated, which is shown in Figure 13.

Based on Figure 13, the external forces computed by Lagrange’s equations and RNEA are close to each other and acceptable when these results are compared with the force sensor. The collision started occurring on the active plate at 3.4 s, and the external force was about ${\left[-0.67-0.69\;3.13\right]}^{T}$ (N). Then, the force reached the maximum value of ${\left[-5.36-2.27\;11.036\right]}^{T}$ (N) at 3.8 s.

The root mean square error (RMSE) is applied to evaluate the accuracy of RNEA, expressed as follows:

(39) $$RMSE=\sqrt{\frac{\sum_{1}^{n}e_{i}^{2}\;}{n}}$$

where $e_{i}$ (i=1,2,…,50) is the error between the computed force by RNEA and the force sensor; n=50 is the number of samples.

RMSE in the X-axis is equal to 0.819 (N), RMSE in the Y-axis is equal to 0.4762 (N), and RMSE in the Z-axis is equal to 1.2823 (N).

### 5.2. Case 2: Collision at an Arm

#### 5.2.1. Collision Detection an Arm

**Figure 14:**
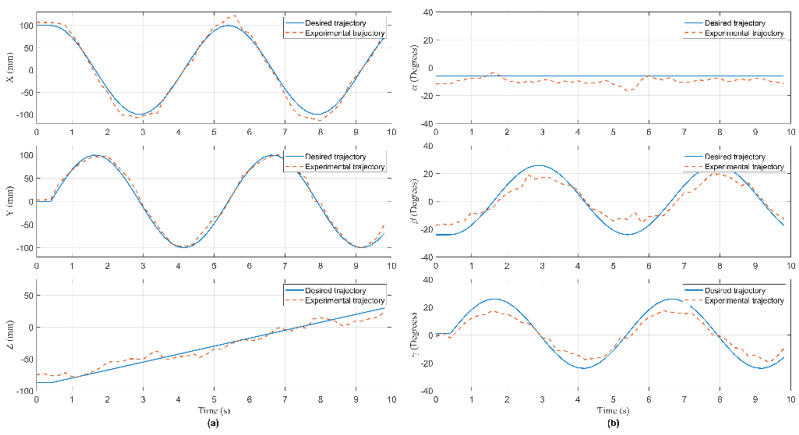
The pose of the end-effector in Case 2: (**a**) The position of the end- effector; (**b**) the orientation of the active plate.

**Figure 15:**
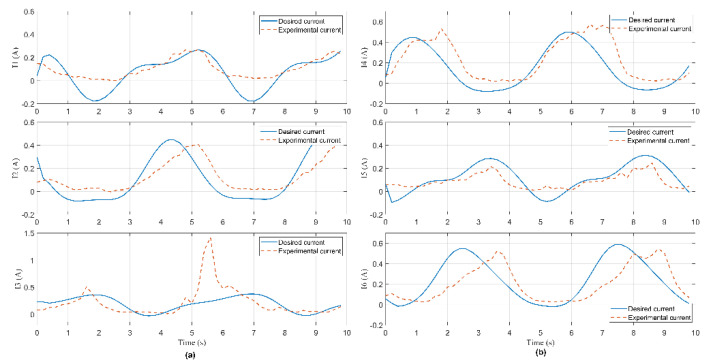
Current signal measured from current sensors in Case 2: (**a**) The 1st, 2nd, and 3rd sensor; (**b**) the 4th, 5th, and 6th sensor.

In this experiment, when the active plate moved in a helix trajectory, a collision occurred due to an external force acting on this arm. The pose of the end-effector is shown in Figure 14. The measured current intensity is shown in Figure 15. Equations of the helix trajectory are presented as follows:

(40) $$\{\begin{array}{cc} x=100\cos\left(0.4\pi t\right)\;\;\; & \left(\mathrm{mm}\right)\\ y=100\sin\left(0.4\pi t\right)\;\;\; & \left(\mathrm{mm}\right)\\ z=-550+5t & \left(\mathrm{mm}\right)\\ \alpha =-6 & \left(\deg\right)\\ \beta =1-25\cos\left(0.4\pi t\right) & \left(\deg\right)\\ \gamma =1+25\sin\left(0.4\pi t\right) & \left(\deg\right) \end{array}$$

**Figure 16:**
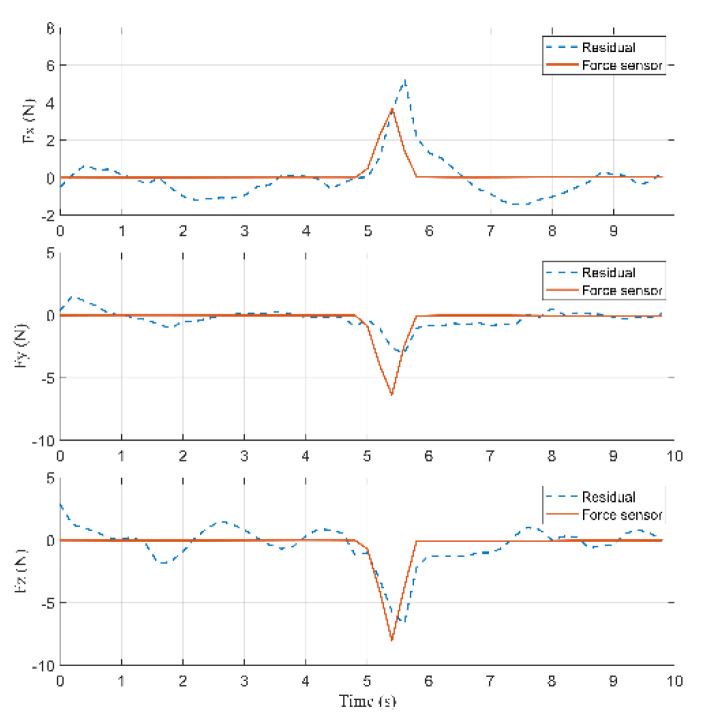
Comparison of the residuals for the force sensor in Case 2.

According to Figure 16, a collision occurred at 5.2 s because the residual in the Z-axis is greater than $r_{low}=2$ (N). The external force reached the maximum value at 5.6 s during the collision.

#### 5.2.2. Collision Isolation an Arm

**Figure 17:**
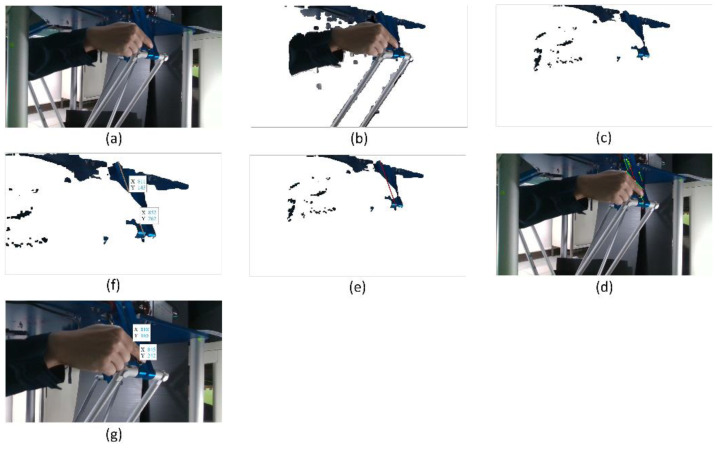
The collision isolation procedure in Case 2: (**a**) The image captured; (**b**) The limited workspace; (**c**) The blue region; (**d**) Straight lines along arms; (**e**) The direction of the arm; (**f**) The intersections of the blue region and the non-blue region in the arm direction; (**g**) The contact point.

When a collision started occurring, no contact point was detected in the image captured from the first camera. The image captured from the second camera at 5.2 s is considered to find out the contact point location, shown in Figure 17a. According to the flowchart of the procedure of collision isolation, shown in Figure 4, objects in this limited workspace are considered in the image captured from the second camera, shown in Figure 17b. Because the color of the arms is blue, the arms can be isolated from the image. An appropriate threshold for the HSV color scale is chosen to extract the blue region in the image, which is shown in Figure 17c. Then, the Canny edge detector and Hough transform are applied to recognize straight-line profiles along arms. These straight lines are shown in Figure 17d. According to the flowchart of the mathematics processing, shown in Figure 5, a fixed pixel is chosen as a motor location where an arm is connected with that motor. According to the fixed pixel and the direction of the arm, all pixels belonging to the blue region in the arm direction are checked to see if any of them intersect the non-blue regions, shown in Figure 17e. The intersections are potential contact point locations. Two intersections should be considered, shown in Figure 17f. The first intersection coordinate is ${\left[145\;811\right]}^{T}$ (pixels), the depth at this point is 440 (mm). This intersection is not the contact point location because a point in the non-blue region has a coordinate equal to ${\left[165\;818\right]}^{T}$ (pixels), and its depth is 379 (mm). The difference between two point’s depth is larger than a chosen depth threshold. The second intersection coordinate is ${\left[262\;850\right]}^{T}$ (pixels), and the depth at this point is 419 (mm). This intersection is the contact point location because a pixel in the non-blue region has a coordinate equal to ${\left[242\;845\right]}^{T}$ (pixel), and its depth is 408 (mm). The difference between two point’s depth is smaller than a chosen depth threshold. The contact point coordinate is identified as ${\left[-113.25\;263.67-111.3\right]}^{T}$ (mm), shown in Figure 17g.

At 5.6 s, a collision occurred on the arm, and the external force reached the maximum value. The intersection coordinate is ${\left[232\;885\right]}^{T}$ (pixels), and the depth at this point is 409 mm. The contact point coordinate is identified as ${\left[-121.99\;278.95-99.66\right]}^{T}$ (mm).

#### 5.2.3. Collision Identification an Arm

**Figure 18:**
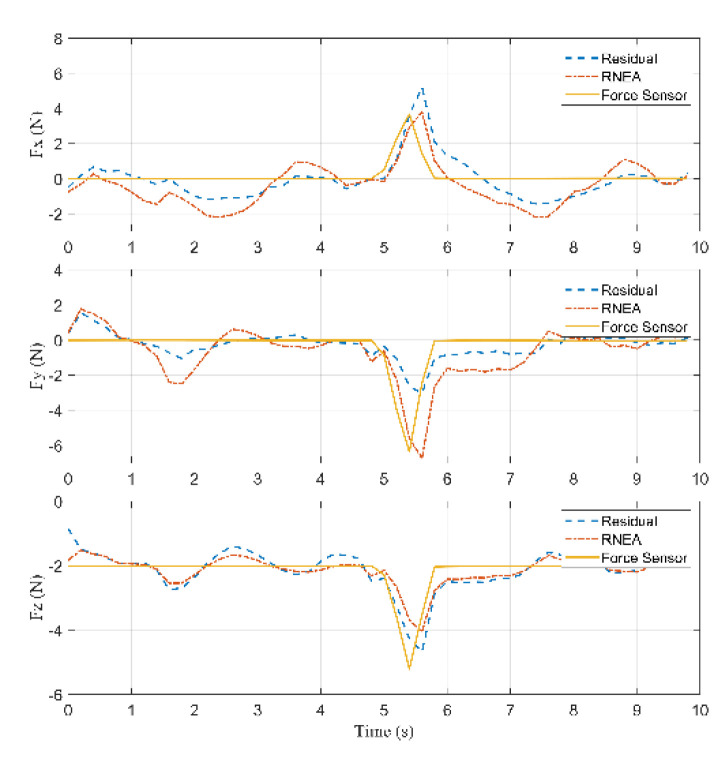
Residuals, the computed forces by RNEA and the force sensor in Case 2.

According to Equation (27), the inertia forces acting on the arms, the rods, and the active plate are calculated. According to Equations (32) and (33), the external force acting on the arm can be calculated, shown in Figure 18.

Based on Figure 18, the collision started occurring on the arm at 5.2 s, and the external force was about ${\left[\begin{array}{ccc} 1.976 & -2.557 & -2.254 \end{array}\right]}^{T}$ (N). Then, the force reached the maximum value of ${\left[\begin{array}{ccc} 4.941 & -7.38 & -5.785 \end{array}\right]}^{T}$ (N) at 5.6 s.

According to Equation (39), root mean square error (RMSE) is applied to evaluate the accuracy of RNEA. RMSE in the X-axis is equal to 1.1193 (N), RMSE in the Y-axis is equal to 1.4974 (N), and RMSE in the Z-axis is equal to 1.1056 (N).

### 5.3. Case 3: Collision at a Rod

#### 5.3.1. Collision Detection at a Rod

**Figure 19:**
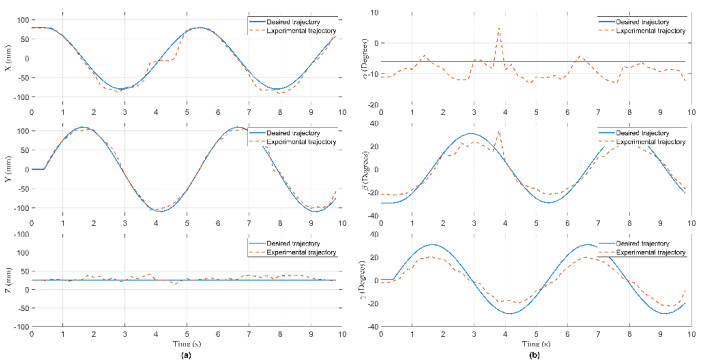
The pose of the end-effector in Case 3: (**a**) The position of the end- effector; (**b**) the orientation of the active plate.

**Figure 20:**
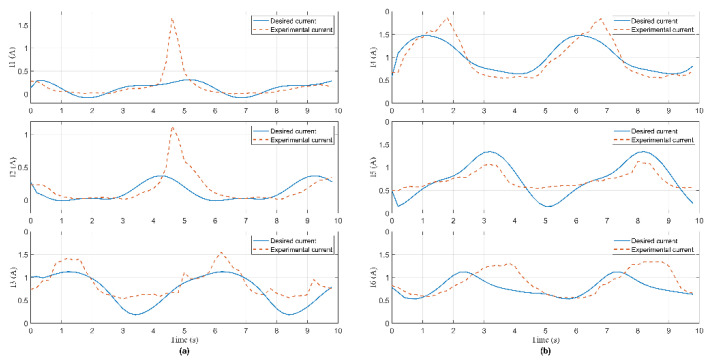
Current signal measured from current sensors in Case 3: (**a**) The 1st, 2nd, and 3rd sensor; (**b**) the 4th, 5th, and 6th sensor.

In this experiment, when the active plate moved in an ellipse trajectory, a collision occurred due to an external force acting on this rod. The pose of the end-effector is shown in Figure 19. The measured current intensity is shown in Figure 20. Equations of the ellipse trajectory are presented as follows:

(41) $$\{\begin{array}{cc} x=80\cos\left(0.4\pi t\right)\;\;\;\; & \left(\mathrm{mm}\right)\\ y=110\sin\left(0.4\pi t\right)\;\;\; & \left(\mathrm{mm}\right)\\ z=-550 & \left(\mathrm{mm}\right)\\ \alpha =-6 & \left(\deg\right)\\ \beta =1-30\cos\left(0.4\pi t\right)\; & \left(\deg\right)\\ \gamma =1+30\sin\left(0.4\pi t\right)\; & \left(\deg\right) \end{array}$$

**Figure 21:**
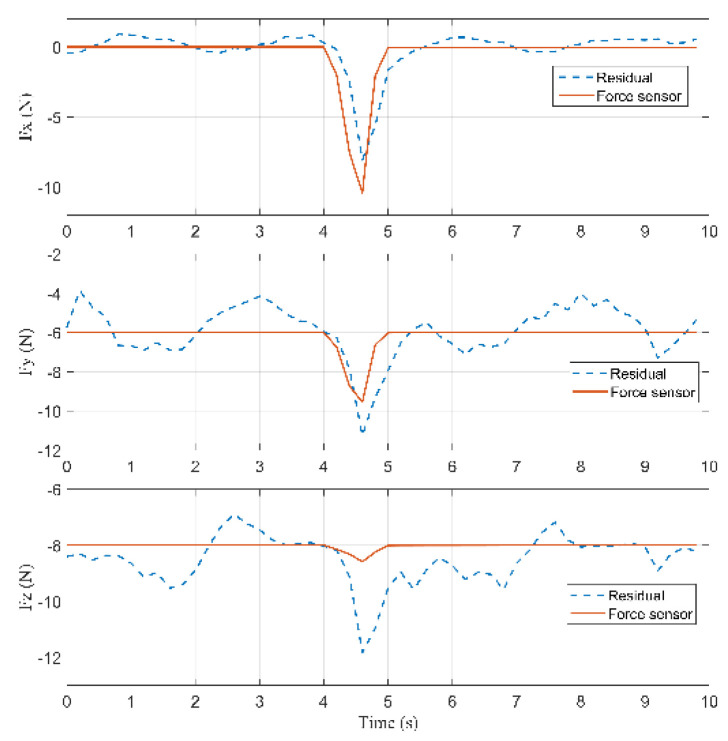
Comparison of the residuals for the force sensor in Case 3.

According to Figure 21, a collision occurred at 4.4 s because the residual in the X-axis is greater than $r_{low}=2$ (N). The external force reached the maximum value at 4.6 s during the collision.

#### 5.3.2. Collision Isolation at a Rod

**Figure 22:**
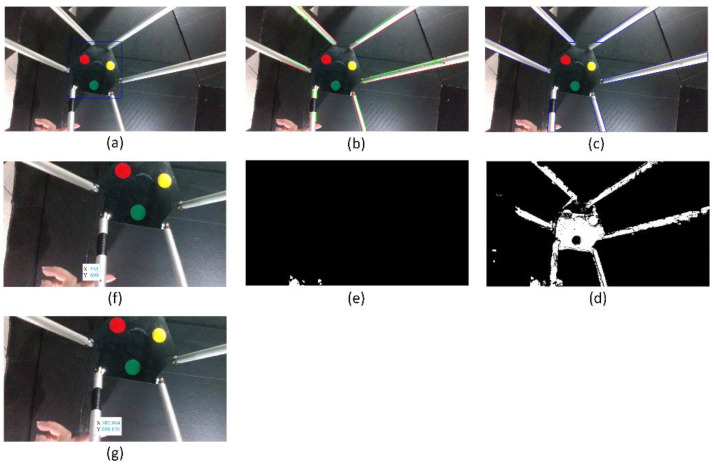
The collision isolation procedure in Case 3: (**a**) The bounding box around the active plate; (**b**) straight lines around rods; (**c**) the recognized rods; (**d**) objects in the workspace; (**e**) the external object; (**f**) potential contact points; (**g**) the contact point.

In a similar method to Case 1, the collision isolation results are shown in Figure 22a–e. According to the flowchart of the mathematics processing shown in Figure 5, 46 midpoints are determined to satisfy the conditions, as shown in Figure 22f. A point represents the above 46 pixels by taking an average. The contact point location is shown in Figure 22 g. Following the strategy mentioned above, the contact point location in the considered image is determined as ${\left[382.8\;698.83\right]}^{T}$ (pixels). According to the camera model, the contact point coordinate is also identified as ${\left[122.93-144.08-411.59\right]}^{T}$ (mm) when a collision occurs at a rod.

At 4.6 s, a collision occurred on the rod, and the external force reached the maximum value. Similarly, 447 pixels are potential contact points on the rod. The contact point location in the considered image is determined as ${\left[399.56\;702.97\right]}^{T}$ (pixels). The contact point coordinate is also identified as ${\left[125.49-137.89-406.5207\right]}^{T}$ (mm) when the external force reaches the maximum value.

#### 5.3.3. Collision Identification at a Rod

**Figure 23:**
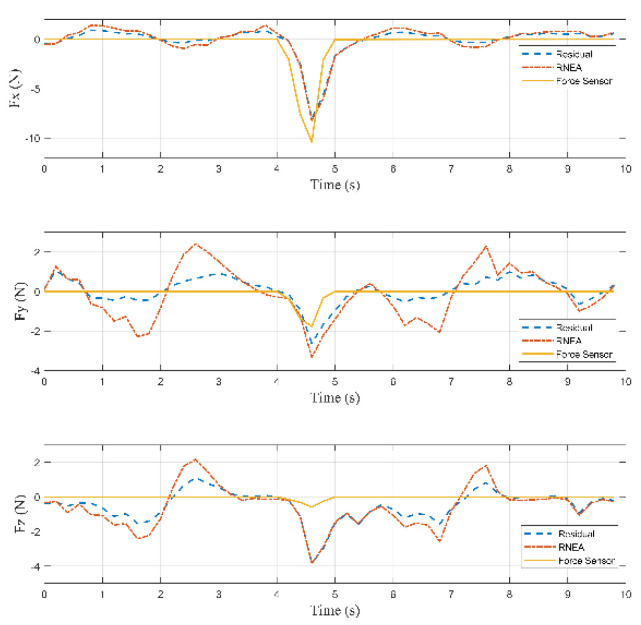
Residuals, the computed forces by RNEA and the force sensors in Case 3.

According to Equation (27), the inertia forces acting on the arms, the rods, and the active plate are calculated. According to Equations (34)–(36), the external force acting on the rod can be calculated, which is shown in Figure 23.

Based on Figure 23, the collision started occurring on the rod at 4.4 s, and the external force was about ${\left[-2.487-0.925-1.121\right]}^{T}$ (N). Then, the force reached the maximum value of ${\left[-8.055-2.631-3.837\right]}^{T}$ (N) at 4.6 s.

According to (39), the root mean square error (RMSE) is applied to evaluate the accuracy of RNEA. RMSE in the X-axis is equal to 1.2268 N, RMSE in the Y-axis is equal to 1.1874 N, and RMSE in the Z-axis is equal to 1.2627 N.

## 6. Conclusions

This study has presented a strategy to solve collision problems for the Hexa parallel robot. The proposed virtual force observer is created by combining the inverse dynamic problem solution and a low-pass filter. When the Hexa robot controls the active plate move with the designed trajectory, the observer monitors and indicates the collision time in the cases of three main parts: active plate, rods, and arms. The multi-dual depth camera system performs effectively for monitoring the workspace and the external obstacle. The contact point location in each collision case is obtained based on the proposed flowchart for the collision phases. The Recursive Newton-Euler algorithm is applied to analyze the dynamics free-body diagram of the Hexa robot. With the determined contact point location, finally, the intensity and directions of the external force are identified based on the Recursive Newton-Euler algorithm. The comparison results for the experimental force sensor show the potential for solving the collision problem in the Hexa robot structure.

## Data Availability

Data available on request due to restrictions of privacy.
